# Angiotensin receptor/neprilysin inhibitor—a breakthrough in chronic heart failure therapy: summary of subanalysis on PARADIGM-HF trial findings

**DOI:** 10.1007/s10741-019-09879-x

**Published:** 2019-11-12

**Authors:** Marcin Książczyk, Małgorzata Lelonek

**Affiliations:** 1 Department of Noninvasive Cardiology, Medical University of Lodz, ul. Żeromskiego 113, 90–549 Lodz, Poland; 2 Department of Interventional Cardiology and Cardiac Arrhythmias, Medical University of Lodz, Lodz, Poland

**Keywords:** Chronic heart failure, Angiotensin receptor/neprilysin inhibitor, Angiotensin-converting enzyme, PARADIGM-HF, Diabetes, Chronic kidney disease, Sudden cardiac death, Quality of life, TRANSITION

## Abstract

It is over 4 years since the Prospective Comparison of angiotensin receptor/neprilysin inhibitor (ARNI) with ACEI to Determine Impact on Global Mortality and Morbidity in Heart Failure (PARADIGM-HF) trial was published in New England Journal of Medicine. The PARADIGM-HF trial was the one that contributed to the official approval to use ARNI simultaneously with cardiac resynchronisation therapy (CRT) or implantable cardioverter-defibrillator (ICD) in patients who receive optimal medical treatment and still presented NYHA II-IV class symptoms according to the 2016 European Society of Cardiology Guidelines for the diagnosis and treatment of acute and chronic heart failure. The aim of this article is to summarise current knowledge on the activity of ARNI in a selected group of patients with heart failure with reduced ejection fraction (HFrEF) based on a recent PARADIGM-HF subanalysis in the field of renal function in patients with and without chronic kidney disease, glycaemia control in patients with diabetes, ventricular arrhythmias and sudden cardiac death and health-related quality of life. This article includes also recently announced findings on the TRANSITION study which revealed that HFrEF therapy with ARNI might be safely initiated after an acute decompensated heart failure episode, including patients with heart failure de novo and ACEI/ARB naïve, both hospitalised or shortly after discharge, in contrary to the PARADIGM-HF trial, where patients had to be administered a stable dose of an ACEI/ARB equivalent to enalapril 10 mg a day for at least 4 weeks before the screening.

## Introduction

It is over 4 years since the Prospective Comparison of ARNI (angiotensin receptor/neprilysin inhibitor—editor’s note) with ACEI (angiotensin-converting enzyme inhibitor—editor’s note) to Determine Impact on Global Mortality and Morbidity in Heart Failure (PARADIGM-HF) trial was published in New England Journal of Medicine. The purpose of the trial was to compare the efficacy of administering enalapril (ACEI) versus sacubitril/valsartan (ARNI) in a selected group of adult patients suffering from heart failure with reduced ejection fraction (HFrEF) of 35% or less and New York Heart Association (NYHA) class II-IV symptoms. The trial was terminated early after a median follow-up of 27 months because of a significantly reduced risk of the primary and secondary endpoint in the sacubitril/valsartan group compared with the enalapril group [1] (Table 1). The PARADIGM-HF trial was the one that contributed to the official approval to use ARNI simultaneously with cardiac resynchronisation therapy (CRT) or implantable cardioverter-defibrillator (ICD) in patients who receive optimal medical treatment and still presented NYHA II-IV class symptoms, according to the 2016 European Society of Cardiology Guidelines for the diagnosis and treatment of acute and chronic heart failure [2]. Glaggett et al. in their study confirmed that life expectancy of patients receiving ARNI might increase from 1 up to 2 years depending on their age compared with patients receiving ACEI [3]. This observation is the ground for strong recommendation to use a combined therapy with sacubitril and valsartan

**Table 1:**
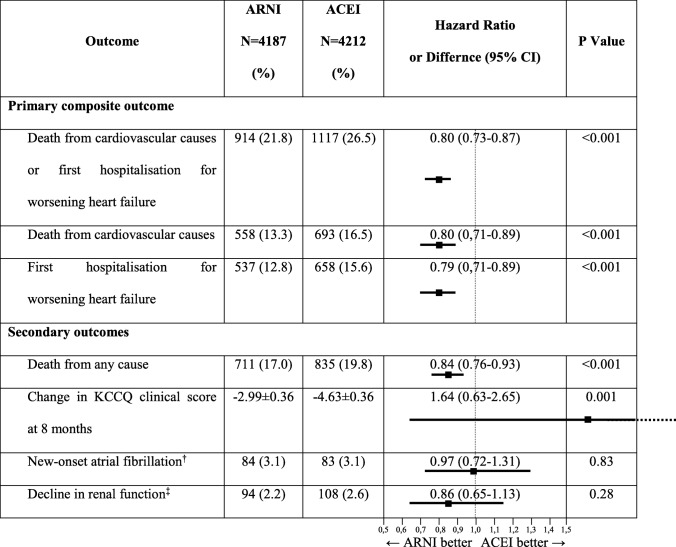
Primary and secondary outcomes for the PARADIGM-HF trial, modified and adapted version [1]

*ACEI* angiotensin-converting enzyme inhibitor (enalapril), *ARNI* angiotensin receptor/neprilysin inhibitor (sacubitril/valsartan), *KCCQ* Kansas City Cardiomyopathy Questionnaire

^†^Atrial fibrillation not presented at randomisation

^‡^End-stage renal disease or a decrease of 50% or more in the estimated glomerular filtration rate (eGFR) from the value at randomisation or a decrease in the eGFR of more than 30 ml/min/1.73 m^2^ to less than 60 ml/min/1.73 m^2^

In the course of time, subsequent analyses of the PARADIGM-HF trial reveal the ARNI effect to be more and more beneficial for patients with heart failure. The aim of this article is to summarise current knowledge on the influence of ARNI in a selected group of patients with HFrEF, based on a recent PARADIGM-HF subanalysis.

**Fig. 1:**
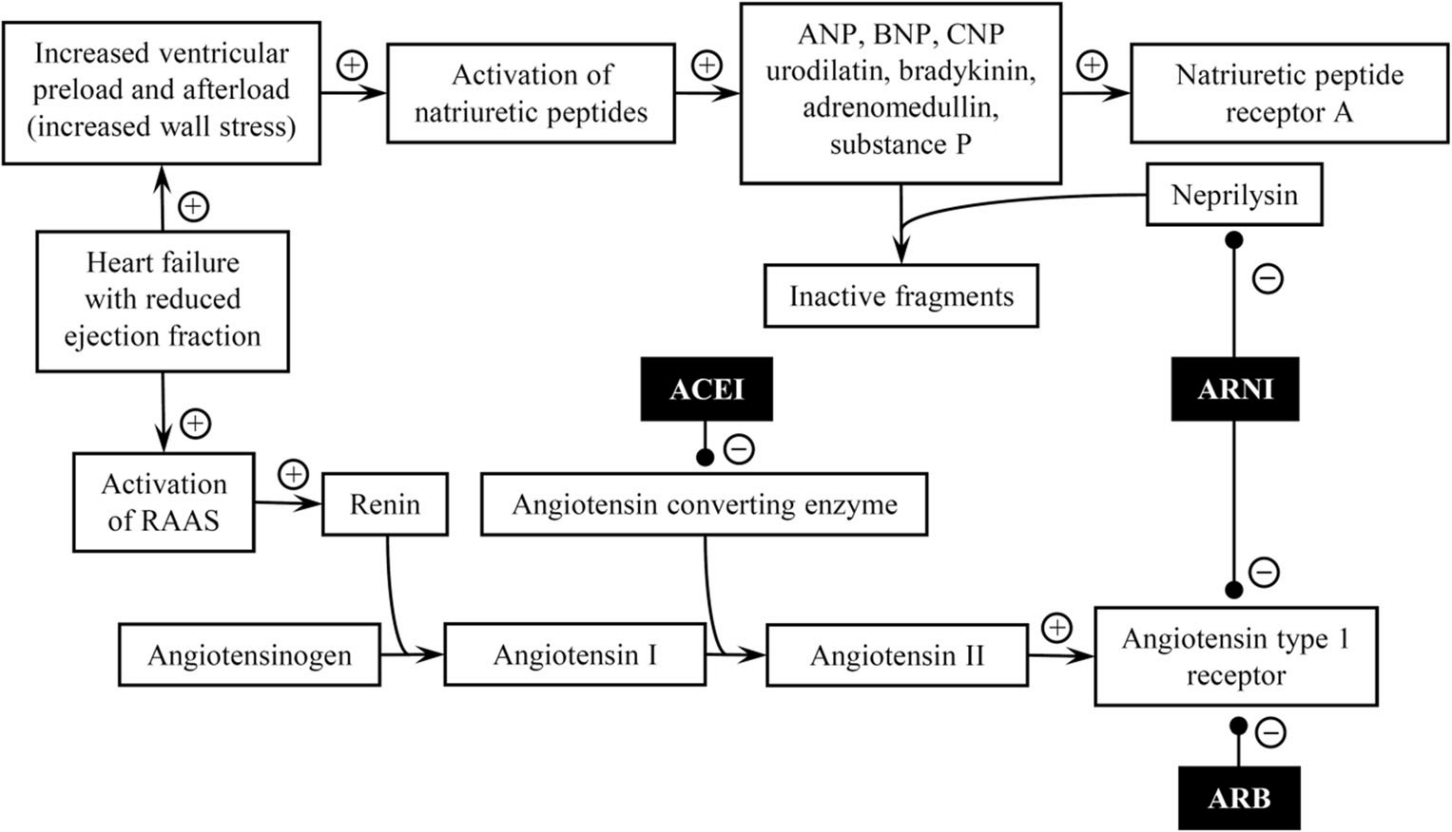
Pathways of activation renin-angiotensin-aldosterone and natriuretic peptide systems and points of interest for ACEI, ARB, and ARNI (“+” for activation and “–” for inhibition); adapted from: Jhund PS, McMurray JJV. *The neprilysin pathway in heart failure: a review and guide on the use of sacubitril/valsartan*. Heart. 2016 Sep 1;102(17):1342-7 [9]. ANP—A-type natriuretic peptide; ACEI—angiotensin-converting enzyme inhibitor; ARB—angiotensin receptor blocker; ARNI—angiotensin receptor/neprilysin inhibitor; BNP—B-type natriuretic peptide; CNP—C-type natriuretic peptide; RAAS—renin-angiotensin-aldosterone system

Randomised controlled trials have proved that the renin-angiotensin-aldosterone system (RAAS) plays an important role in the pathophysiology of heart failure, thus the morbidity and mortality of patients with HFrEF may be improved by blocking RAAS [4, 5]. In patients with HFrEF, upregulation of RAAS occurs, which in turn leads to excessive production of natriuretic peptides: B-type natriuretic peptide (BNP), atrial-derived A-type natriuretic peptide (ANP), endothelium-derived C-type natriuretic peptide (CNP) and kidney-derived urodilatin. In consequence, natriuretic peptides modulate the response to RAAS by promoting natriuresis and vasodilatation [6, 7]. It seems that the best strategy to improve outcomes in HFrEF would be inhibition of breakdown of the natriuretic peptides and blocking the RAAS at the same time [8, 9]. Neprilysin is a metalloendopeptidase and cleaves several different substrates such as ANP, BNP, CNP, endothelin, substance P, bradykinin and angiotensin I-II to inactive fragments, and as a consequence, it reduces the serum levels all of these peptides [9–11]. Inhibition of neprilysin with sacubitril results in an increase in serum levels of both natriuretic peptides and angiotensin II which stimulates the RAAS activity and counteracts the beneficial activity of natriuretic peptides [12]. Combination of sacubitril—neprilysin inhibitor—and valsartan—angiotensin receptor inhibitor—seems to be a better option than any other drug administered in heart failure management as it affects the pathophysiology of heart failure: it prevents degradation of natriuretic peptides and inhibits RAAS at the same time (Fig. 1) [9, 12]. Myhre et al. showed that during the treatment with ARNI, the serum BNP concentration increased up to 2–3 folds during the first 8–10 months compared to the initial BNP concentration while serum concentration of N-terminal prohormone of BNP (NT-proBNP) was relatively stable and its increase was not so dramatic as it was for BNP; an increase in the BNP concentration, accompanied by an increase in the NT-proBNP level, was associated with worse outcomes [13]. Nasrien et al. conducted a study on 23 subjects with HFrEF to assess the impact of sacubitril/valsartan on the level of natriuretic peptides, other than BNP in the serum with the use of different tests. It was revealed that the ANP concentration increased up to 2 folds by the first follow-up visit after on average 22 days of the treatment with ARNI, whereas the change in the CNP concentration remained inconsistent [14]. It is still unclear if the ARNI effect relies more on ANP or BNP activity, as BNP is a relatively poorer substrate for neprilysin than ANP or even CNP [15–17].

## Effects of ARNI on renal function in patients with and without chronic kidney disease

Brenner et al. showed that RAAS inhibition reduces urinary albumin excretion and slows down the progression to end-stage renal disease, especially in patients without chronic kidney disease (CKD) or diabetes [18]. Treatment with RAAS inhibitors becomes limited in patients with renal impairment or diabetes because the risk of serum creatinine increase or hyperkalemia is greater than in patients with HFrEF and without the abovementioned co-morbidities [19].

A subanalysis of the PARADIGM-HF trial, performed by Damman et al., concerning the effects of ARNI on renal function in comparison with ACEI, shows superiority of ARNI over ACEI both in patients with and without CKD [20]. At the screening, the eGFR in the trial was 70 ± 20 ml/min/1.73 m^2^ and 2745 patients of 8399 had CKD; the median urinary albumin/creatinine ratio (UACR) was 1.0 mg/mmol (UACR was available in 1872 patients). The rate of decline in eGFR was significantly lower in the sacubitril/valsartan than in the enalapril arm (− 1.61 ml/min/1.73 m^2^/year; [95% CI, − 1.77 to − 1.44] *vs* − 2.04 ml/min/1.73 m^2^/year [95% CI, − 2.21 to − 1.88], *p* < 0.001), despite a greater increase in UACR in the sacubitril/valsartan than in the enalapril arm (1.20 mg/mmol [95% CI, 1.04 to 1.36] *vs* 0.90 mg/mmol [95% CI, 0.77 to 1.03], *p* < 0.001). The results were similar in patients with and without CKD at screening (Table 2). Although the decrease in eGFR was similar in both groups and the incidence of the pre-specified renal outcome (*a* ≥ 50% decrease in eGFR or *a* > 30 ml/min/1.73 m^2^decrease in eGFR to < 60 ml/min/1.73 m^2^ or an end-stage renal disease) was similar between patients with or without CKD at screening, a post hoc analysis of a conventional renal composite outcome (an end-stage renal disease or *a* ≥ 50% decrease in the eGFR) revealed that end-stage renal disease occurred significantly less frequently in patients treated with sacubitril/valsartan, irrespective of presence of CKD at screening. What is more, the increase in UACR was associated with a higher risk of the pre-specified composite renal endpoint only in the enalapril group, but not in the sacubitril/valsartan group.

**Table 2:**
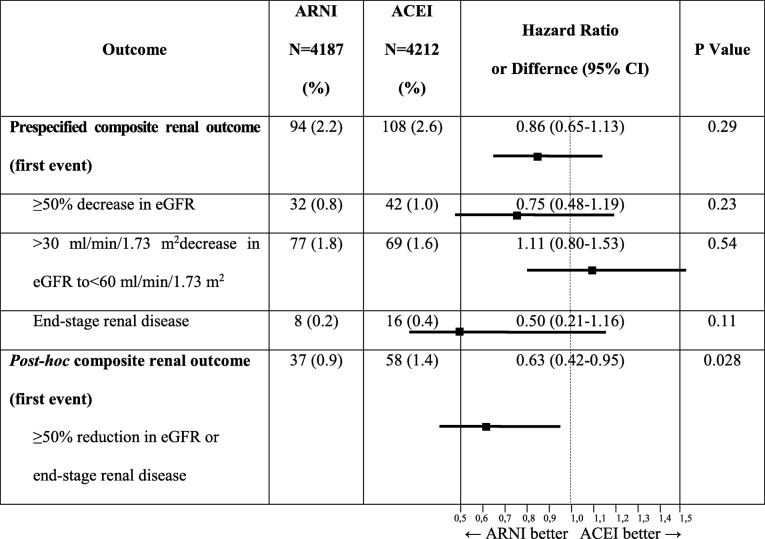
Renal endpoints for PARADIGM-HF trial; modified and adapted version [20]

*ACEI* angiotensin-converting enzyme inhibitor (enalapril), *ARNI* angiotensin receptor/neprilysin inhibitor (sacubitril/valsartan), *eGFR* estimated glomerular filtration rate

Packer et al. analysed again the PARADIGM-HF trial in the context of deterioration of renal function in patients with (3784 patients) and without concomitant diabetes (4615 patients). The deterioration of renal function was significantly lower in patients without diabetes than in diabetic patients (− 1.0 ml/min/1.73 m^2^/year [95% CI, − 1.2 to − 1.0] *vs* − 2.0 ml/min/1.73 m^2^/year [95% CI, − 2.1 to − 1.9], *p* < 0.0001) irrespective of the used treatment agent [21]. According to Packer et al., the rate of decline in eGFR was lower in the sacubitril/valsartan than in the enalapril arm but the statistically obtained values differ from those obtained by Damman et al. because of different determined criteria (− 1.3 ml/min/1.73 m^2^/year *vs* − 1.8 ml/min/1.73 m^2^/year, *p* < 0.0001). It is pointed out that the benefit of using ARNI was greater in diabetic than in non-diabetic patients (difference 0.6 ml/min/1.73 m^2^/year [95% CI, 0.4–0.8] *vs* 0.3 ml/min/1.73 m^2^/year [95% CI, 0.2–0.5], *p* = 0.038).

ARNI, apart from a decrease of lessening of the eGFR, also reduces the risk of hyperkalemia in patients who take mineralocorticoid receptor antagonists (MRAs) together with RAAS inhibitors. That was proved in a subanalysis conducted by Desai et al. [22]. The authors compared 4671 patients who were taking MRAs at baseline and who were randomly assigned to the group of sacubitril/valsartan or enalapril. Although the total number of incidences of hyperkalemia, identified for potassium level > 5.5 mEq/l, was similar in both treatment groups, incidences of severe hyperkalemia, identified for potassium level > 6.0 mEq/l, were more frequent in the enalapril than in the sacubitril/valsartan group (3.1/100 patient/year *vs* 2.2/100 patient/year; HR 1.37 [95% CI, 1.06–1.76], *p* = 0.02) (Table 3). An analysis of 791 patients with no MRAs at baseline who started taking MRAs during the PARADIGM-HF trial showed that severe hyperkalemia was more common in the group of enalapril than in the group of sacubitril/valsartan (3.3/100 patient/year *vs* 2.3/100 patient/year; HR 1.43 [95% CI, 1.13–1.81], *p* = 0.003). Although the risk of hyperkalemia between groups was not statistically significant, the risk of severe hyperkalemia was statistically significant, both for MRA non-recipients and MRA recipients [23]. Generally, patients treated with MRAs at baseline were younger, demonstrated more severe HF symptoms and higher potassium levels and used diuretics more frequently. It seems therefore that ARNI might attenuate the risk of hyperkalemia in patients with HF in the course of MRA therapy.

**Table 3:**
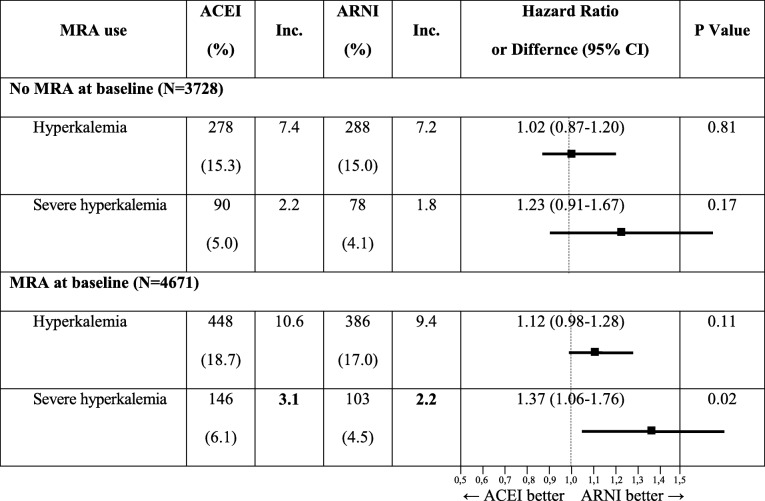
Prevalence of hyperkalemia and severe hyperkalemia in patients with and without MRAs at baseline assigned to the arms of PARADIGM-HF trial; modified and adapted version [22]

*ACEI* angiotensin-converting enzyme inhibitor (enalapril), *ARNI* angiotensin receptor/neprilysin inhibitor (sacubitril/valsartan), *MRA* mineralocorticoid receptor antagonist, *Inc.* incidence: no of cases/100 patients/year

The PARADIGM-HF study had several limitations, which makes it necessary to analyse the results particularly carefully. Firstly, patients were not randomised according to MRA use at baseline. Secondly, those who received MRAs were not monitored for dosing or compliance with MRAs during the study [22, 23]. Finally, the run-in period excluded patients who did not tolerate ARNI or ACEI or developed hyperkalemia during its administration. Due to residual differences between MRA-treated and MRA-naïve groups, some patients were excluded during the initial period of the trial. The true risk of hyperkalemia in both the groups randomised to the enalapril or sacubitril/valsartan arm may be underestimated and the hyperkalemia risk and the activity of ARNI, associated with the above risk, requires further investigation [22, 24].

## Effects of ARNI on glycaemia control in patients with diabetes

Diabetes mellitus is a comorbidity frequently accompanying chronic heart failure and is an independent risk factor for heart failure progression [25, 26]. Findings of Kristensen et al. reveal that approximately 1 in 5 patients with HFrEF without a confirmed history of diabetes suffers from undiagnosed diabetes and 1 patient in 3 has actually pre-diabetes. The authors have divided all patients without history of diabetes into 3 groups, according to the concentration of glycated heamoglobin (HbA1c), measured at screening: normal < 6.0%, pre-diabetes 6.0–6.4% (2103 patients) and undiagnosed diabetes ≥ 6.5% (1106 patients); patients diagnosed previously with diabetes (2907 patients) were considered to have diabetes irrespective of HbA1c concentration [27]. An analysis showed that patients with a history of diabetes had a higher risk of the primary composite outcome of hospitalization due to heart failure or cardiovascular mortality, compared with those without a history of diabetes (HR 1.38 [95% CI, 1.25 to 1.52], *p* < 0.001); a higher risk was also observed in patients with undiagnosed diabetes and diagnosed diabetes, compared with those with normal HbA1c (HR 1.39 [95% CI, 1.17–1.64], *p* < 0.001 *vs* HR 1.64 [1.43–1.87], *p* < 0.001, respectively) and patients with pre-diabetes compared with those with normal HbA1c (HR 1.27 [95% CI, 1.10–1.47], *p* < 0.001).

**Fig. 2:**
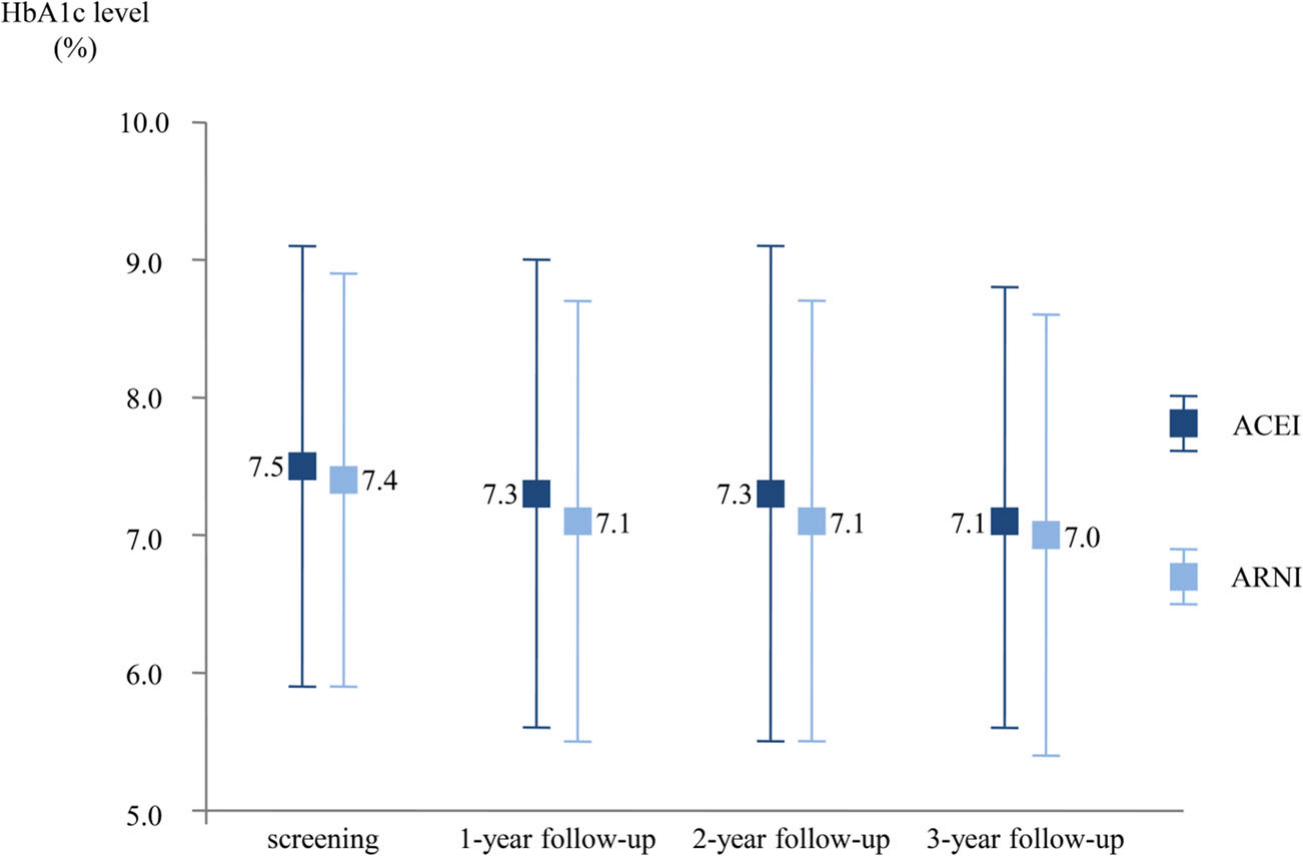
Concentration of glycated haemoglobin at screening and over 3 years of follow-ups in patients with diagnosed diabetes and non-diabetic patients and HbA1c concentration ≥ 6.5% at screening randomised to enalapril or sacubitril/valsartan arm [21]. ACEI—angiotensin-converting enzyme inhibitor; ARNI—angiotensin receptor/neprilysin inhibitor; HbA1c—glycated haemoglobin

ARNI improves glycaemia control in patients with previously diagnosed diabetes and those with undiagnosed diabetes and HbA1c concentration ≥ 6.5% at screening that was shown by Seferovic et al. in their post hoc analysis [26]. Although no significant differences in HbA1c levels between randomised arms at screening were observed, HbA1c concentrations were lowered by 0.16% (± 1.4) in the enalapril arm while 0.26% (± 1.25) in the sacubitril/valsartan arm during the first year of follow-up (between-group reduction 0.13% [95% CI, 0.05–0.22], *p* = 0.0023) and remain lower over the 3-year follow-up (between-group reduction 0.14% [95% CI, 0.06–0.23], *p* = 0.0055) (Fig. 2). Additionally, new use of insulin or oral antihyperglycaemic drugs was lower in the group of sacubitril/valsartan than in the group of enalapril, which enhances the position of ARNI in optimal medical treatment of patients with HFrEF and concomitant diabetes.

**Fig. 3:**
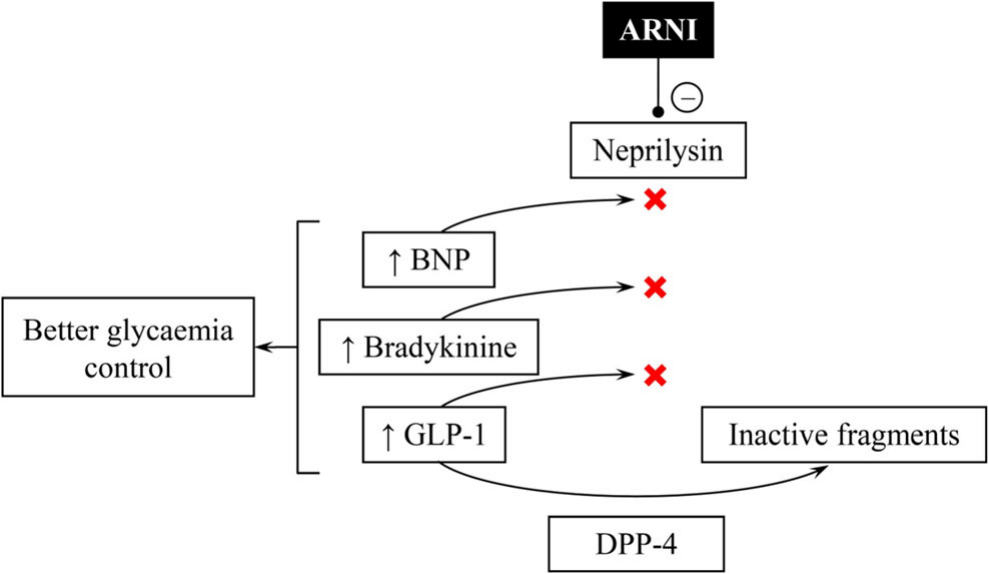
Mechanism of better glycaemia control when the use of sacubitril/valsartan underlies inter alia an increased concentration of peptides which cannot be degraded by actually blocked neprilysin (“–” for inhibition, “↑” for increase). ARNI—angiotensin receptor/neprilysin inhibitor; BNP—B-type natriuretic peptide; DPP-4—dipeptidyl peptidase-4; GLP-1—glucagon-like peptide-1

The pathophysiological mechanisms, underlying better glycaemia control when ARNI is administered, are not fully understood and need further investigation. Currently, it is usually attributed to higher concentration of active peptides which could not be degraded by inactive neprilysin like BNP, bradykinin and glucagon-like peptide-1 (GLP-1) (Fig. 3) [28–33]. Although the use of sacubitril/valsartan seems to be beneficial in patients with diabetes by potentiating antihyperglycaemic acting of endogenous GLP-1, the use of ARNI in concomitant treatment with GLP-1 long-acting analogues may be harmful in patients with both diabetes and heart failure. A deleterious effect of co-administering ARNI and GLP-1 analogues is explained by augmentation of GLP-1 influence on a heart rate and a concentration of cyclic adenosine monophosphate which limited ARNI efficacy [34].

## Effects of ARNI on all-cause mortality including sudden cardiac death

The impact of administration of sacubitril/valsartan on decrease in the number of cardiovascular deaths and all-cause mortality compared with enalapril in patients with chronic heart failure in the PARADIGM-HF trial, is incontestable. Desai et al. examined the data from the trial to better understand the mode of death [35]. According to the authors, most of the deaths were due to cardiovascular disorders (80.9%), and among them, the majority of cases were sudden cardiac deaths (44.8%) followed by worsening heart failure (26.5%) [35]. The risk of cardiovascular death was significantly reduced in the sacubitril/valsartan arm (HR 0.80 [95% CI, 0.72–0.89], *p* < 0.001) compared with enalapril and in deaths due to cardiovascular reasons; the reduction in mortality with sacubitril/valsartan was greater in comparison to enalapril and similar for both sudden cardiac deaths (HR 0.80 [95% CI 0.68–0.94], *p* = 0.008) and deaths due to increasing heart failure (HR 0.79 [95% CI 0.64–0.98], *p* = 0.034).

The precise mechanism of reduced mortality and sudden cardiac death in the group of sacubitril/valsartan remains unclear. However, Sarrias and Bayes-Genis suggest a direct involvement of neprilysin inhibition in the process [36]. Increase in natriuretic peptide concentration leads to molecular cascade, which in turn, reduces cardiac inflammation, myocyte death, hypertrophy and fibrosis; all these 4 factors are beneficial for patients with HFrEF as they reverse or reduce left ventricular remodeling. By inducing an antiarrhythmic effect with enkephalins, endorphins and bradykinin, they reduce the rate of ventricular tachyarrhythmias and ventricular premature beats which results in a decline of the rate of sudden cardiac death.

The beneficial effects of ARNI application, compared with ACEI on reduction of appropriate shocks, non-sustained ventricular tachycardia (nsVT) and premature ventricular contraction (PVC), were shown by de Diego et al. in a prospective, non-randomised trial which included 120 patients with HFrEF and implanted ICDs who were initially treated with ACEI or ARB within the first 9 months and then ACEI or ARB was subsequently changed to ARNI for the next 9 months of the follow-up [37]. ARNI, compared with ACEI or ARB, significantly decreased: nsVT episodes (5.4 ± 0.5 *vs* 15 ± 1.7, *p* < 0.002), sVT and appropriate ICD shocks (0.8% *vs* 6.7%, p < 0.02), PVCs per hour (33 ± 12 *vs* 78 ± 15, *p* < 0.0003) and increased biventricular pacing percentage (from 95% ± 6% to 98.8% ± 1.3%, *p* < 0.02). Such types of original studies provoke questions whether we still need ICDs if we have ARNI [38], but the answer is nowadays not obvious and the potential antiarrhythmic activity of ARNI needs further studies.

## Effects of ARNI on health-related quality of life

The design of the PARADIGM-HF trial included not only objective and measureable data collection but also but also allowed patients to make a subjective analysis of their health-related quality of life (HRQL) when they used ARNI or ACEI, evaluated with the Kansas City Cardiomyopathy Questionnaire (KCCQ) completed, at randomisation and after 4, 8, 12, 24 and 36 months [39]. KCCQ includes both question about clinical status (i.e. extremities swelling, fatigue or shortness of breath) and social or physical activity (i.e. showering/bathing, hurrying/jogging or working/doing household chores).

This subanalysis performed by Lewis et al. showed that KCCQ clinical summary scores and KCCQ overall summary scores at 8 months were better in patients treated with sacubitril/valsartan compared with those treated with enalapril (+ 0.64 *vs* − 0.29, *p* = 0.008, for clinical summary scores and + 1.13 *vs* − 0.14, *p* < 0.001, for overall summary scores, respectively) [40].

**Fig. 4:**
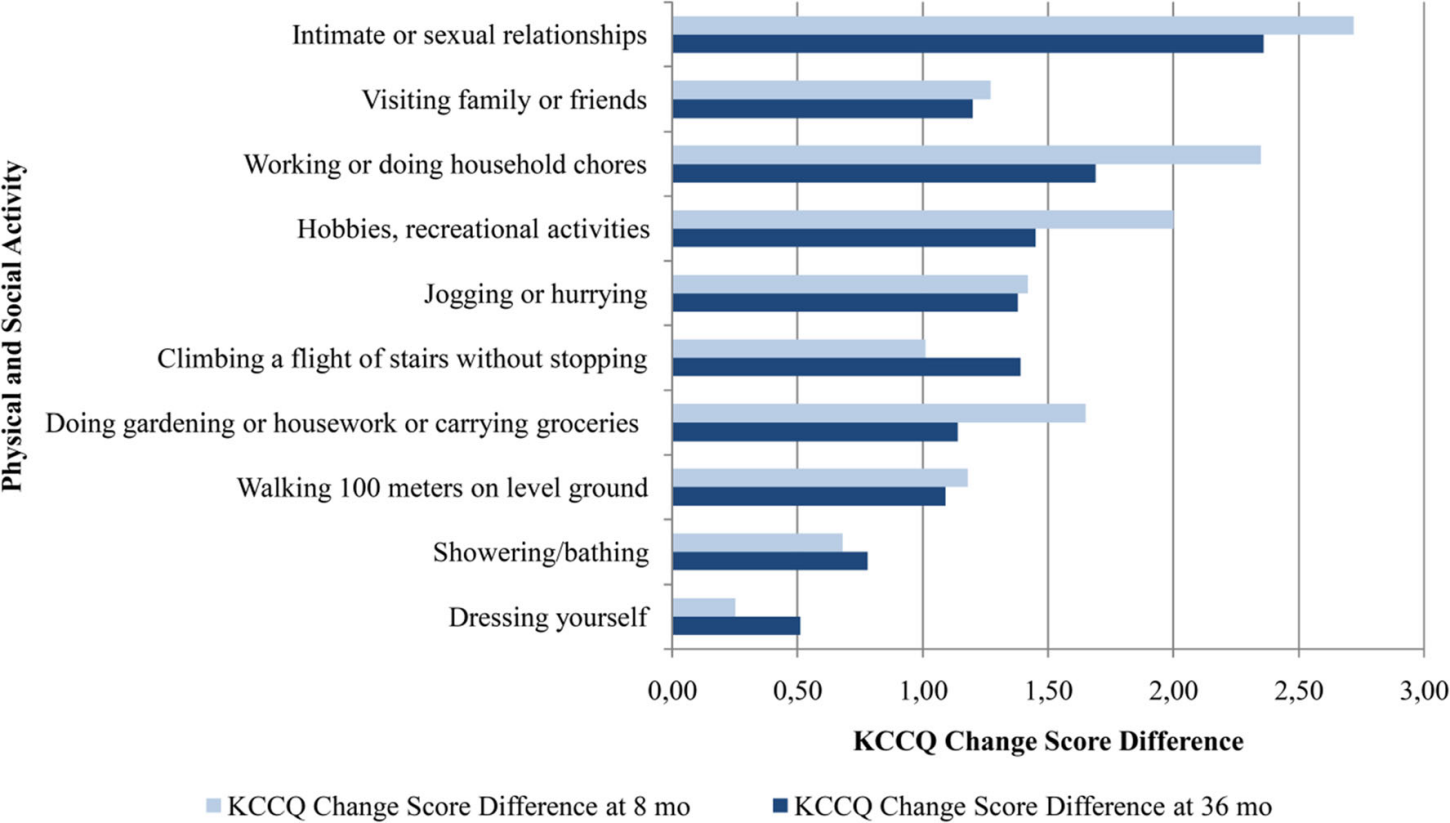
Change score differences of KCCQ physical and social activities between enalapril and sacubitril/valsartan group at 8-month and at 36-month follow-up [37, 38]. KCCQ—Kansas City Cardiomyopathy Questionnaire; mo—months

Similarly, Chandra et al. compared adjusted change scores in most physical and social activities at 8 months and during 36 months [41]. It is suggested that such types of activity like jogging and sexual relationships had the lowest mean scores whereas getting dressed and showering had the highest mean scores at baseline. The analysis performed at 8 months, reported significant adjusted change score in all social or physical activities except for getting dressed and showering (0.25 [95% CI, − 0.66–1.16], *p* = 0.59, and 0.68 [95% CI, − 0.24–1.59], *p* = 0.15, respectively), with the largest adjusted change score in household chores and sexual relationships (2.35 [95% CI, 1.19–3.50], *p* < 0.001, and 2.72 [95% CI, 0.97–4.46], *p* = 0.002, respectively) in the group of patients treated with sacubitril/valsartan (Fig. 4); the results were similar after 36 months. As a result, the improvement in both physical and social activity is claimed to be circa 9 years of aging (95% CI, 4–13 years, *p* < 0.001) in favour of sacubitril/valsartan arm.

## When is the moment for ARNI initiation? TRANSITION study

TRANSITION (Comparison of Pre- and Post-discharge Initiation of LCZ696 Therapy in HFrEF Patients After an Acute Decompensation Event) is a randomised, simultaneous, open-label study which aimed at comparing pre- and post-discharge (1–14 days) initiation of sacubitril/valsartan in patients with HFrEF, LVEF ≤ 40% and NYHA class II–IV following hemodynamic stabilisation (defined as no need for intravenous diuretics in 24 h prior to screening and systolic blood pressure > 110 mmHg for at least 6 h prior to randomisation), after an episode of acute decompensated heart failure (ADHF), including patients with newly diagnosed HF and also ACEI/ARB naïve (no ACEI/ARB for ≥ 4 weeks before hospitalisation). In contrast, all patients of PARADIGM-HF were administered a stable dose of an ACEI/ARB equivalent to enalapril 10 mg/day for at least 4 weeks before the screening.

The primary endpoint was the proportion of patients achieving 200 mg sacubitril/valsartan twice a day at 10 weeks’ post-randomisation, and it was reached by 45.0% of 493 patients in the pre-discharge arm and 50.4% of 490 patients in the post-discharge arm (relative risk ratio [RRR] 0.893; [95% CI, 0.783–1.019], *p* = 0.092) [42, 43].

As the incidence of adverse events of sacubitril/valsartan and their discontinuations was similar in pre- and post-discharge groups, it is concluded that HFrEF therapy with ARNI might be safely initiated after an ADHF episode, including patients with new-onset and ACEI/ARB naïve, both in-hospital or shortly after discharge.

## Conclusions

The PARADIGM-HF was a well-designed clinical trial that confirmed benefits due to initiation of sacubitril/valsartan in patients diagnosed with HFrEF, LVEF ≤ 35% and NYHA II–IV symptoms despite optimal medical treatment accompanied by cardiac resynchronisation therapy (CRT) or implantable cardioverter-defibrillator (ICD). Although, we do not understand all mechanisms of ARNI activity and the way it decreases HF symptoms, some of the subanalyses provide us invaluable experience on how ARNI reduce the decline in eGFR and risk of hyperkalemia in patients with and without chronic kidney disease, improve glycaemia control in diabetic patients, prevent ventricular arrhythmias or sudden cardiac death and ameliorate health-related quality of life. The new TRANSITION study results, where sacubitril/valsartan is initiated earlier than previously, suggest that ARNI will be used more frequently soon. It brings a hope for patients, affected by HF and who have not responded really positively to a conventional therapy.
